# Metasurface Reflector (MSR) Loading for High Performance Small Microstrip Antenna Design

**DOI:** 10.1371/journal.pone.0127185

**Published:** 2015-05-27

**Authors:** Md Rezwanul Ahsan, Mohammad Tariqul Islam, Mohammad Habib Ullah, Mandeep Jit Singh, Mohd Tarmizi Ali

**Affiliations:** 1 Department of Electrical Electronic and Systems Engineering, Faculty of Engineering and Built Environment, Universiti Kebangsaan Malaysia (UKM), Bangi, Selangor, Malaysia; 2 Department of Electrical Engineering, Faculty of Engineering, University of Malaya (UM), Kuala Lumpur, Malaysia; 3 Antenna Research Group (ARG), Microwave Technology Centre (MTC), Faculty of Electrical Engineering (FKE), Universiti Teknologi Mara (UiTM), Shah Alam, Selangor, Malaysia; Glasgow University, UNITED KINGDOM

## Abstract

A meander stripline feed multiband microstrip antenna loaded with metasurface reflector (MSR) structure has been designed, analyzed and constructed that offers the wireless communication services for UHF/microwave RFID and WLAN/WiMAX applications. The proposed MSR assimilated antenna comprises planar straight forward design of circular shaped radiator with horizontal slots on it and 2D metasurface formed by the periodic square metallic element that resembles the behavior of metamaterials. A custom made high dielectric bio-plastic substrate (*ε*_*r*_ = 15) is used for fabricating the prototype of the MSR embedded planar monopole antenna. The details of the design progress through numerical simulations and experimental results are presented and discussed accordingly. The measured impedance bandwidth, radiation patterns and gain of the proposed MSR integrated antenna are compared with the obtained results from numerical simulation, and a good compliance can be observed between them. The investigation shows that utilization of MSR structure has significantly broadened the -10dB impedance bandwidth than the conventional patch antenna: from 540 to 632 MHz (17%), 467 to 606 MHz (29%) and 758 MHz to 1062 MHz (40%) for three distinct operating bands centered at 0.9, 3.5 and 5.5 GHz. Additionally, due to the assimilation of MSR, the overall realized gains have been upgraded to a higher value of 3.62 dBi, 6.09 dBi and 8.6 dBi for lower, middle and upper frequency band respectively. The measured radiation patterns, impedance bandwidths (*S11*<-10 dB) and gains from the MSR loaded antenna prototype exhibit reasonable characteristics that can satisfy the requirements of UHF/microwave (5.8 GHz) RFID, WiMAX (3.5/5.5 GHz) and WLAN (5.2/5.8 GHz) applications.

## Introduction

In recent days, the microstrip patch antennas of low-profile, inexpensive, compact, multiband, easy and simple design structure have gained considerable research attention due to their effective utilization in developing portable wireless communication devices [1]. The antenna as a vital component of any communication module that resides at the center of the state-of-the-art of multi-functionalities, it is always requested to design a multiband antenna with wide bandwidth, adequate gain and stable radiation patterns to integrate with the modern multi-purpose wireless devices [2]. Although, the microstrip patch antennas are excellent contestant to serve the functions, however narrow bandwidth, inadequate radiation properties and inappropriate gain function of the conventional microstrip patch antennas restrict them from various wireless communication systems. To alleviate the said limitations, the researchers are enforced to search alternative means for achieving optimum performance from a single antenna module. Recent literatures suggest various promising techniques to overcome the shortcomings of patch antenna associated with bandwidth, gain and radiation efficiencies. Most of the techniques offer the performance enhancement in exchange of complicated structures or methods like array technique, stacking antenna elements in vertically or horizontally, applying magnetic dielectric, engineering the radiating or ground plane, cavity coupled procedure, inclusion of parasitic elements, partial reflective surface, utilization of metamaterial or metasurface structures [3–8].

The most frequent and typical technique to achieve high gain out of the antenna is to use multiple elements as an array which are excited through properly designed feeding network. However, great complicacy is there with the design of feed structure for obtaining desired phase response which may lead to signal loss and unstable radiation performance [5]. Application of electromagnetic coupling through stacking/multi layering/air gap coupling is another conventional way to obtain wide bandwidth and enhanced gain. However, this method increases the overall antenna volume and difficult to construct low profile antenna [3]. Some researchers have also investigated the utilization of partial reflective surface for improving the antenna performance. For such technique, it is reasonably required to maintain a minimum of λ/2 (in terms of resonant frequency) Fabry-Perot resonant cavity between the metallic ground plane and reflective surface. Moreover, this technique provides very low bandwidth due to the preservation of high quality factor which however restrict the antenna from many applications [9]. Although, the cavity gap can be reduced up to λ/4 by utilizing the artificial magnetic conductor and considering the ground as perfect magnetic conductor, the aperture efficiency appears low and the thickness problem may not be possible to mitigate completely [10]. The concept of metamaterial and/or metamaterial inspired 2D surfaces (simply metasurface) have already started to emerge as a new technique for improving the overall performance of microstrip patch antennas.

Following the theoretical analysis of Veselago [11], Pendry [12], it was Smith who first physically constructed a composite microwave medium which constitutes periodic arrays of split-ring resonators and metallic wires, then experimentally verified the anomalous refraction due to the negative refraction in the artificially engineered materials, termed simply metamaterials [13]. Since then, a numerous research work has been done in conjunction with the novel ideas, suggestions, theories related to the artificial fabrication of the electromagnetic and optical properties of the materials for a variety of applications. In metamaterials, the engineering manipulation of wave properties may contribute substantial size and weight reduction of modules, devices, and systems together with performance enhancements. Metamaterials have experienced a rapid growth over the past few years and new capabilities are being explored to broaden the range of unique electromagnetic properties for functional devices, including tunable, switchable, and nonlinear properties [14]. The planar version of 3D bulk metamaterial is simply termed as metasurface which has the advantages of taking less space and offer low loss, effectively utilized in several ways to improve the basic antenna features as bandwidth, gain, directivity, radiation patterns [15,16]. The metamaterial incorporated surfaces are responsible for producing the reflection on the incident field depending on the geometrical structures of the region. As a consequence, the concept of variation in the printed metamaterial structures as geometrical shapes and their arrangements on dielectric have contributed to the change of impedance, which may find promising applications for the designing of microwave antennas and devices. The extensive reviews of recently published articles have revealed several approaches associated with metamaterials/metasurfaces to enhance antenna performances in terms of gain and/or bandwidth and some cases only applicable for single frequency band [4,7,17–19]. In addition to that, the reported antennas may have deficiencies in size, cost, and multiplex geometric structures which further lead to search better solution for achieving improved antenna performances while maintaining considerably compact size, planar, simple and easy to fabricate geometrical structure.

In search of finding the solutions for compact structure, we are proposing high dielectric ceramic filled bioplastic sandwiched substrate (*ε*_*r*_ = 15) for maintaining the compactness of the antenna and the enhancement of antenna performance by integrating the MSR structure. In this paper, the performances of circular shape horizontal slotted patch antenna have been investigated through the metasurface reflector (MSR) loading. By utilizing the partial ground plane, meandered stripline and MSR structure, the antenna is capable to meet the specification of UHF/microwave RFID bands [20], WiMAX 3.5/5.5 bands [21] and WLAN 5.2/5.8 bands [22] while maintaining considerably smaller in size. A thorough study has been carried out and details of the operational explanations are added for the triple band MSR embedded patch antenna with numerical and experimental result analysis. The pairs of symmetrical cutting slots loaded on the circular radiating patch generate the resonant frequency as expected for RFID and WiMAX/WLAN bands, whereas the meandered-strip-line feed and partial ground plane assist in widening the operating bands. The metasurfaces inspired by 3D metamaterial have been widely investigated by the researchers to simultaneously improve the antenna bandwidth and gain [4,8,17,23–25]. However, the improvements are not adequate to cover multiple operating bands by maintaining compactness, simple geometry, and cost effectiveness. Therefore, we intended to maximize the enhancement of antenna bandwidth and gain simultaneously by embedding MSR on the opposite side of radiating patch. The proposed MSR embedded antenna differs from some of the most relevant reported antennas [4,8,17] in terms of performance and design geometry. More specifically, the proposed antenna with MSR structure has got comparatively smaller dimension and offers wider bandwidth and higher gain. The simulation results are well comparable and closely interlinked with the experimental results thus validate the application of MSR for enhancing the functionalities of the antenna. The final outcome of the designed MSR loaded patch antenna is planar, having a low profile with no-via. Thereupon, the simple photolithography based fabrication technique can be used to manufacture the prototype of the antenna and MSR structure. The proposed antenna with MSR structure has been fabricated with their optimum parameters and performance criteria are analyzed. The experimental results of the antenna loaded with MSR structure represent good impedance matching for RFID and WiMAX/WLAN bands, satisfactory radiation efficiency, and noticeable high gain for the operating bands; all of properties reasonably make the proposed MSR incorporated patch antenna suitable for RFID and WiMAX/WLAN communication.

## Complete Design Structure of the Antenna

The designing of the MSR integrated multiband patch antenna includes: the geometrical structure of the antenna (radiating patch and the ground plane) and the architecture of the MSR, which are discussed below with details parametric investigations.

### A. Design Concept of MSR Loaded Antenna Geometry

**Fig 1:**
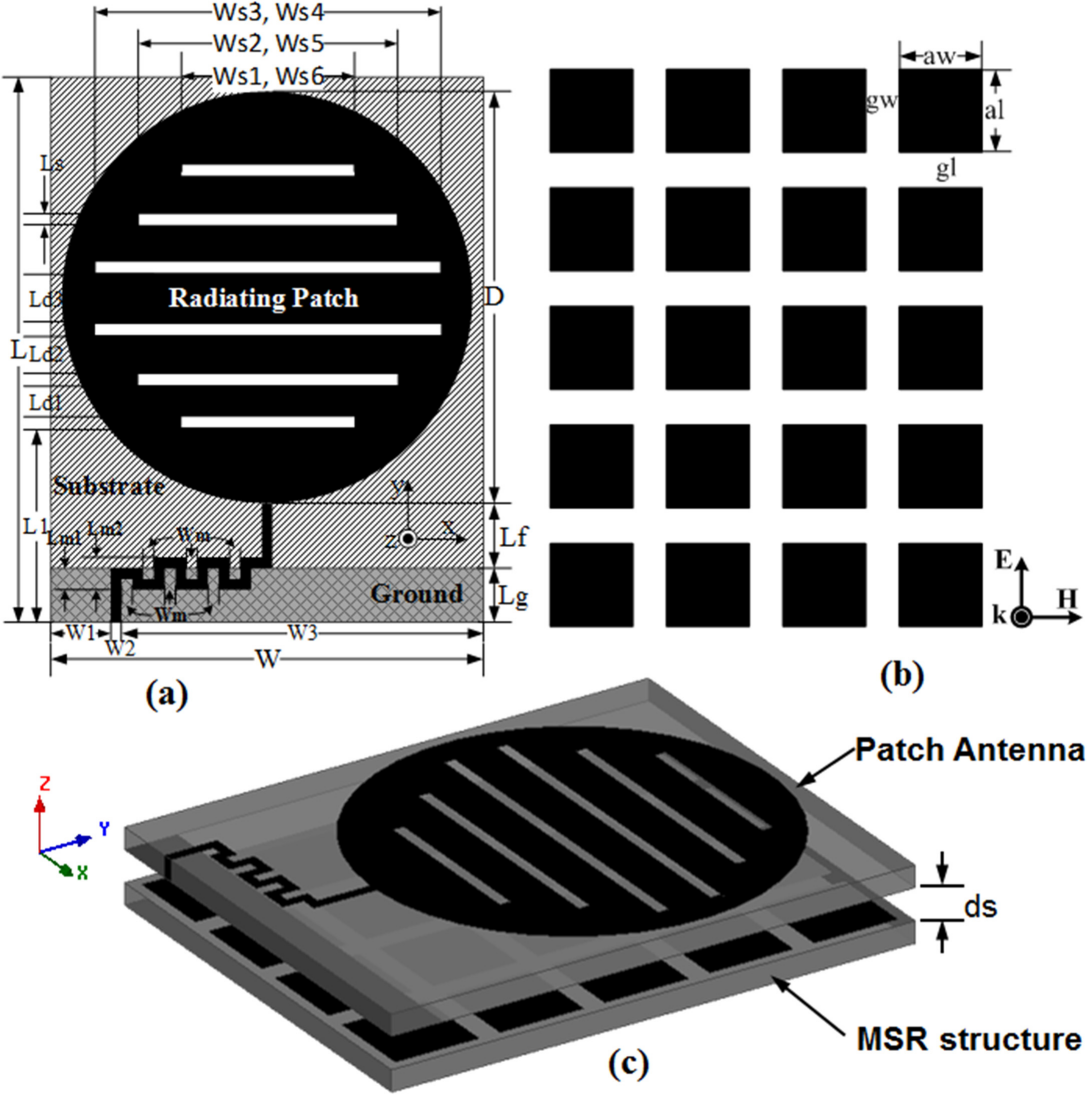
Geometry of the (a) patch antenna (b) MSR structure and (c) MSR loaded patch antenna.

The detailed geometrical configuration of the proposed MSR integrated antenna is depicted in Fig 1 through conventional patch antenna, MSR structure and conceptual design of the MSR loaded patch antenna respectively. The circular/disc shape slotted patch antenna has been considered in this work as numbers of researchers have considered the structure with slot loading due to stable EM radiation property and simple design scheme [26–29]. Before selecting the patch geometry, the elementary dimension of the rectangular radiator based on the fundamental operating frequency is calculated by using the widely popular transmission line model [30]. The custom-made bioplastic substrate of dielectric constant *ε*_*r*_ = 15, loss tangent *tanδ* = 0.002 and thickness *h* = 2.0 mm is chosen for this research. On the basis of fundamental frequency (*f*_*1*_), dielectric constant of substrate (*ε*_*r*_) and thickness of the substrate (*h*), free space velocity of light (c) and wavelength (λ), the mathematical formulae can be derived as:

$$\text{Effective dielectric constant},\;\varepsilon_{r.ef}=\frac{\varepsilon_{r}+1}{2}+\frac{\varepsilon_{r}-1}{2}{\left(1+\frac{10h}{W}\right)}^{-0.5}$$ (1)

$$\text{Fringing length},\;\Delta L=0.412\frac{(\varepsilon_{r.ef}+0.3)\left[\frac{W}{h}+0.264\right]}{(\varepsilon_{r.ef}-0.258)\left[\frac{W}{h}+0.8\right]}h$$ (2)

$$\text{Based on fundamental frequency},f_{1}=\frac{c}{2(L+2\Delta L)\sqrt{\varepsilon_{r.ef}}},$$ (3)

$$\text{the patch length},\;L=0.5\frac{\lambda }{\sqrt{\varepsilon_{r}}}\text{-}2\Delta \text{L and patch width},\;W=\frac{\lambda }{2}\sqrt{\frac{\varepsilon_{r}+1}{2}}$$ (4)

However, the mathematical formulation is valid only for solid rectangular patch without any slot inscribes on the metallic radiator surface. By wisely selecting the patch antenna dimension in accordance with the calculated one, numerous iterative simulations have to be carried out by employing full-wave EM simulator. In this research work, the finite element method (FEM) based 3D full-wave EM field solver HFSS is utilized throughout various simulations. Beside the complete numerical simulation with given geometrical parameters of the antenna and material properties, the built-in Optimetrics engine in HFSS can be used to obtain optimum parametric dimension based on specified criteria. However, proper selection of feeding method, fine meshing of the structure, appropriate EM boundary conditions and more importantly the right choice of driven solution (based on solution frequency and frequency sweep) are essentially required to obtain converged solution for the specified design.

On the basis of the literatures [31–35], slot pairs are embedded on the circular patch radiator for achieving multiband functionality. During the design process, the estimated length (*L*) and width (*W*) of the antenna kept constant while the diameter (*D*) of the patch was arbitrarily tuned to obtain best impedance matching for the specified operating frequencies. As the slot pairs have substantial effect on the antenna performances (reflection coefficient, resonance and bandwidth), their dimensions and positions are considered for detailed parametric analyses to find out best solution. To attain adequate wide bandwidth, the meandered feeding technique and partial ground plane are utilized; numerically analyzed for best antenna performances and are discussed in subsequent section. To get the utmost performance from the optimized design of the microstrip patch antenna, further extensive investigation has been carried out by integrating an MSR structure (Fig 1(B)) at the bottom of the antenna (Fig 1(C)). The separation distance between the patch antenna and MSR structure are also considered for parametric investigation and optimized distance has been considered in the physical prototype. All of the dimensional antenna parameters are listed in Table 1 with initial value and final optimized value (obtained by using Optimetrics in HFSS). The rest of the antenna parameters (which are not considered for parametric analysis) are estimated by performing several exploratory simulations and kept invariant throughout the simulation processes.

**Table 1:** Parametric values of the proposed MSR embedded multiband antenna as depicted in Fig 1.

Parameter	Initial value (mm)	Optimized value (mm)	Parameter	Initial value (mm)	Optimized value (mm)
*L*	51	51	*W*	41	41
*L1*	20	20	*W1*	7	7
*Ld1*	2.5	2.5	*W2*	1	1
*Ld2*	3	3	*W3*	33	33
*Ld3*	3.5	3.5	*Wm*	1	1
*Ls*	0.5	1.5	*D*	41	38
*Lf*	6	6	*Lg*	51	5
*Lm1*	2	2	*gw*	2	2
*Lm2*	3	3	*aw*	8	8
*Ws1*, *Ws6*	10	18	*gl*	2	2
*Ws2*, *Ws5*	10	24	*al*	8	8
*Ws3*, *Ws4*	10	32	*ds*	0	4

### B. Parametric Analysis

The proposed MSR assimilated triple band antenna is fed by optimized meander stripline and backed by partial ground plane. On the basis of design formulations and processes, the optimal dimensions of the radiating patch, size and position of the slots, dimensions of the ground plane, placement of MSR are obtained from parametric investigations. Consider the prior knowledge that some of the antenna parameters (i.e. size of antenna, feed position) and performance criteria (i.e. gain, radiation property) have serious effect on the microstrip patch, hence these are not directly considered during the parametric investigations. The most influential design parameters of the antenna (number of slot pairs, feeding technique, dimension of ground plane and separation distance between the patch antenna and MSR structure) are analyzed by using the full-wave EM simulator to obtain the optimized value.

#### (i) Analysis of Patch Antenna Structure

**Fig 2:**
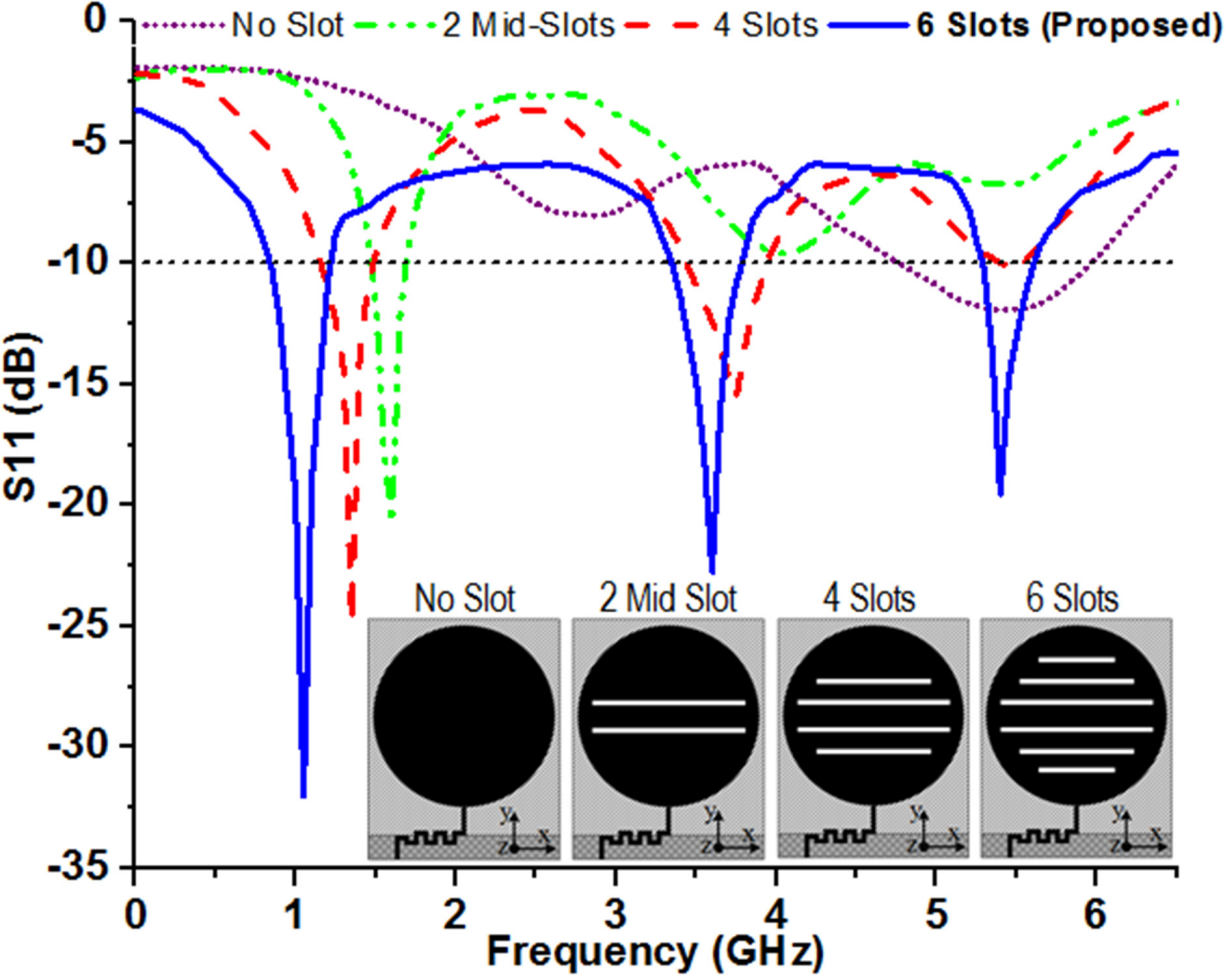
Effect of inserting different numbers of slots on the radiating patch.

For the design of the proposed antenna, one of the considerations is multi-resonant functionality which is achieved through slot loading on the patch. The idea of slot antenna first proposed by Alan Blumlein and its functionality is based on the Babinet’s principle [36,37]. The technique of cutting slots on the radiating patch is effectively utilized to reduce the size of the patch antenna and create multiple frequency band within the desired frequency range by wisely selecting the dimension and position of the slot [31–35]. The horizontal slots on the radiating surface enable variation in current flow and direction which in turn changes the impedance of the patch to produce a desired resonant mode. The simulated frequency response of the scattering parameter (S-parameter), *S11* of the antenna for the insertions of slots is shown in Fig 2. Insertion of two middle slots (*Ws3*, *Ws4*) which are mirrored along the center of the radiating patch introduces the lower resonance frequency centered at 1.58 GHz. Etching out the other two horizontal slots (*Ws2*, *Ws5*) symmetric along the center are accountable for generating second resonant frequency at around 3.74 GHz while forcing the lower resonance towards the lower band as expected. Inclusion of the outer slots (*Ws1*, *Ws6*) significantly affect the surface current distribution on the radiating element and thus help the patch antenna to resonate at three distinct frequencies centered at 1.04 GHz, 3.6 GHz and 5.4 GHz. The arrangement and mirrored placement of the slots effectively extend the streamlines of the current associated with the fundamental mode of resonances and results in lower down the resonant frequencies in comparison to the antenna without any slot.

**Fig 3:**
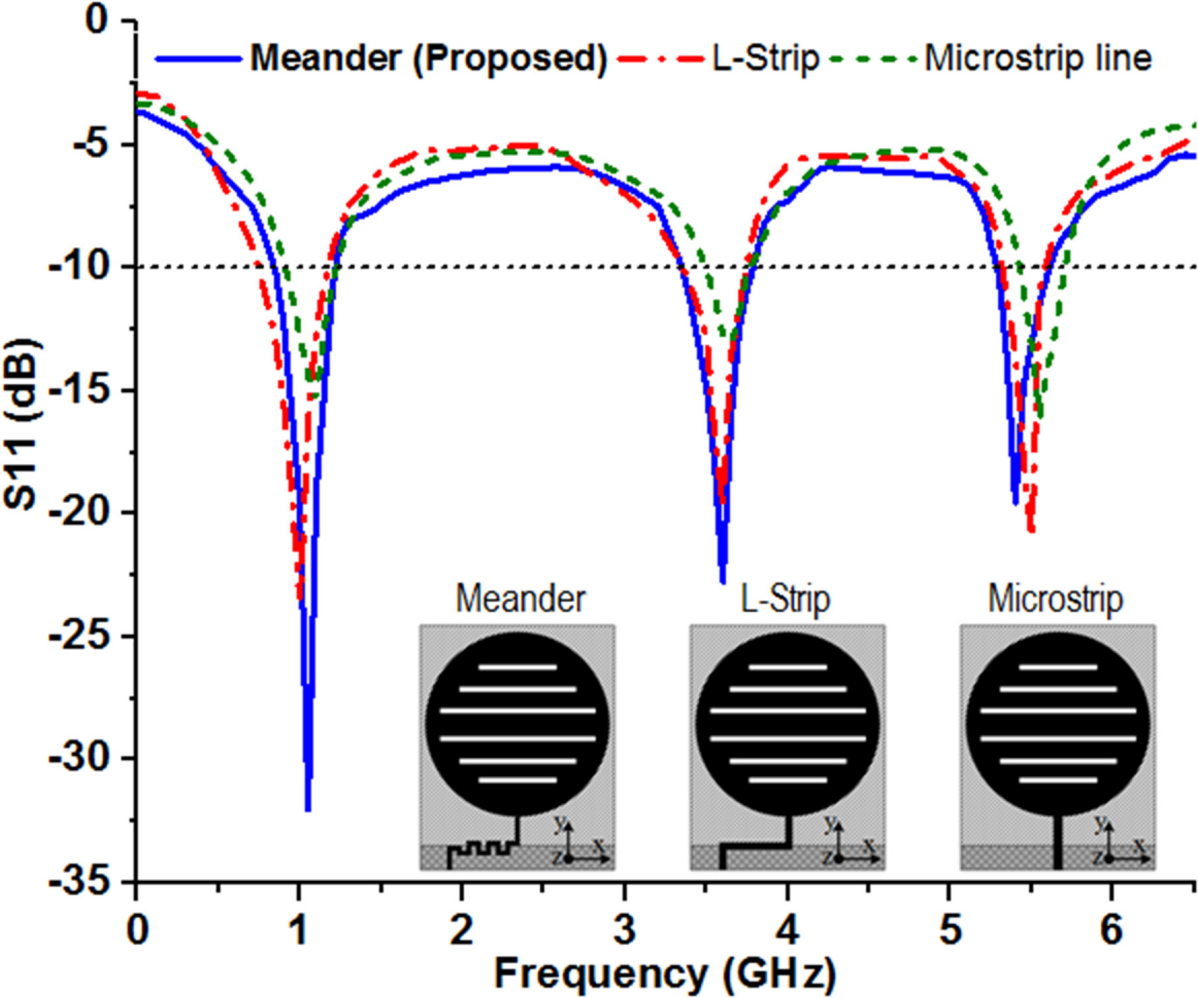
Response of different feeding techniques on the S-parameter, *S11*.

The low antenna profile is preserved by using high permittivity (*ε*_*r*_ = 15), low loss (*tan δ* = 0.002), thickness (*h* = 2.0 mm), ceramic filled composite bio-plastic custom dielectric substrate for the designing of the proposed antenna. The high dielectric substrate has been prepared by following the standard processes; contains three layers of sandwiched structure of 1.5 mm thick ceramic and 0.25 mm thick bioplastic layers on both sides. The biodegradable bioplastic sheet is prepared from locally available biomass organic source (substances from palm and cornstarch) that can replace the conventional petroleum-based plastics materials for high dielectric substrate preparation [38]. Due to the use of high dielectric substrate, the bandwidth of the antenna is certainly reduced [39] which is overcome by utilizing the bandwidth enhancement techniques namely meandered stripline feed and partial ground plane [40]. The meander stripline feed structure helps to reduce the input impedance mismatch. Fig 3 shows that the meandered stripline feed structure has achieved much wider bandwidth than the conventional L-strip and microstrip feed. The meander strip-line feed structure has contributed to good impedance matching and hence leads to the performance enhancement in terms of bandwidth and S-parameter, *S11*.

**Fig 4:**
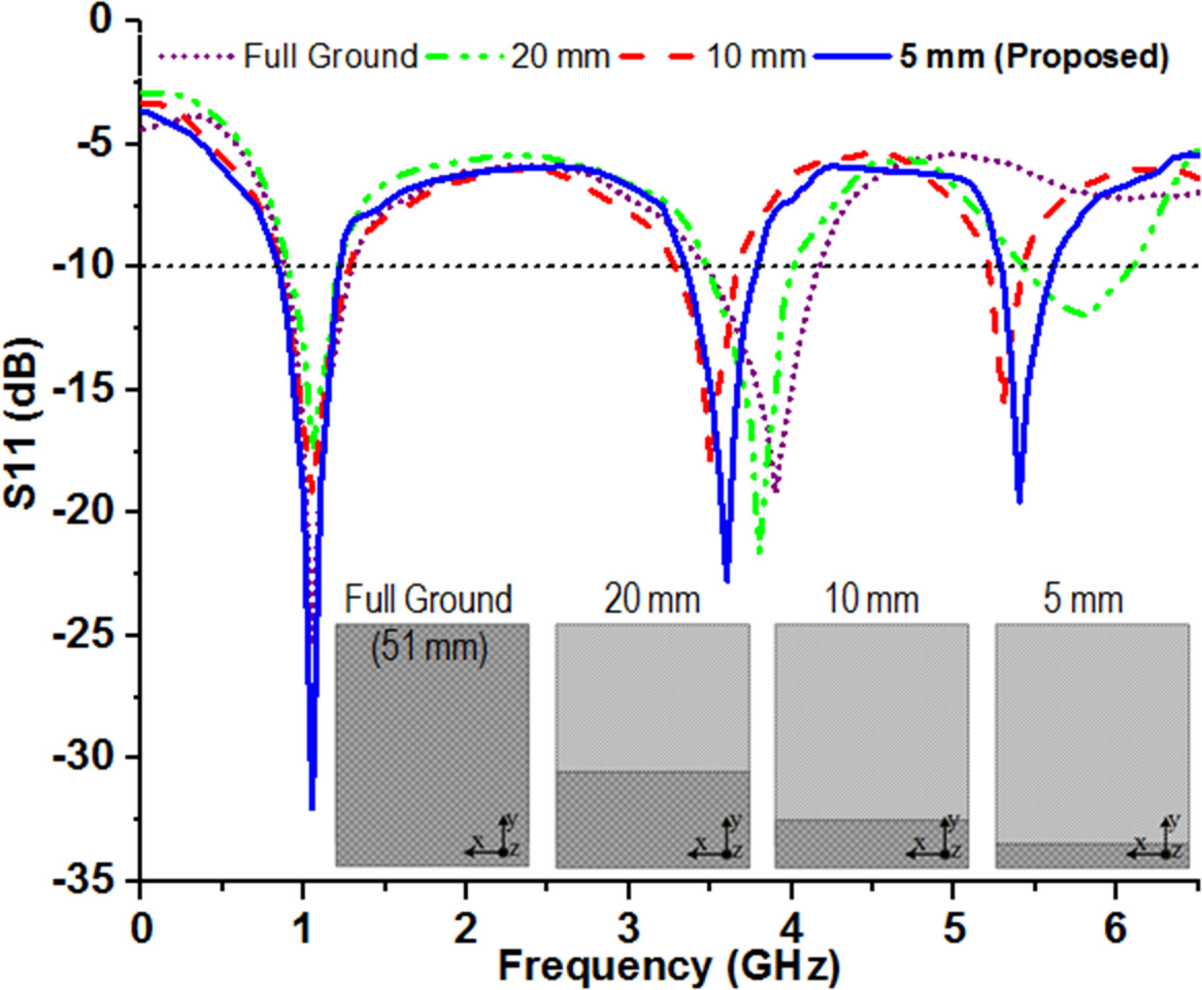
Effect of the dimension of ground plane on the S-parameter, *S11*.

The dimension of ground plane has a dominant effect on the mode of excitation and hence the working bandwidth of the antenna. It has already been analyzed by the researchers that, utilization of defected/partial ground plane would certainly increase the bandwidth of the antenna in the cost of increased cross-polar effect and back lobe radiation due to the absence of PEC (perfect electric conductor) ground [41,42]. The utilization of MSR structure reflects back the radiated wave which further accumulated with the incident wave to give rise in bandwidth. The parametric study has explored for optimized ground plane length of 5.0 mm in which case the desired resonant responses and bandwidths are obtained as shown in Fig 4. The antenna is powered through a standard 50 ohm SMA interface connector whose central conductor is soldered to the meandered stripline feed connected to the radiating patch and the outer conductor is soldered to the ground plane. The perfection of the soldering process has been confirmed through a continuity test for both the radiating patch and the ground plane with the SMA connector. Employment of the optimal partial ground plane instead of full length (51.0 mm) has contributed performance degradation, lead to the trade-off between antenna dimension, S-parameter, operating bandwidth and gain. For achieving better antenna performance other than the conventional design, the concept of MSR is applied, which is placed below the designed antenna and the geometrical planar structure is presented in Fig 1 (C).

#### (ii) Analysis of Metasurface Reflector Structure

**Fig 5:**
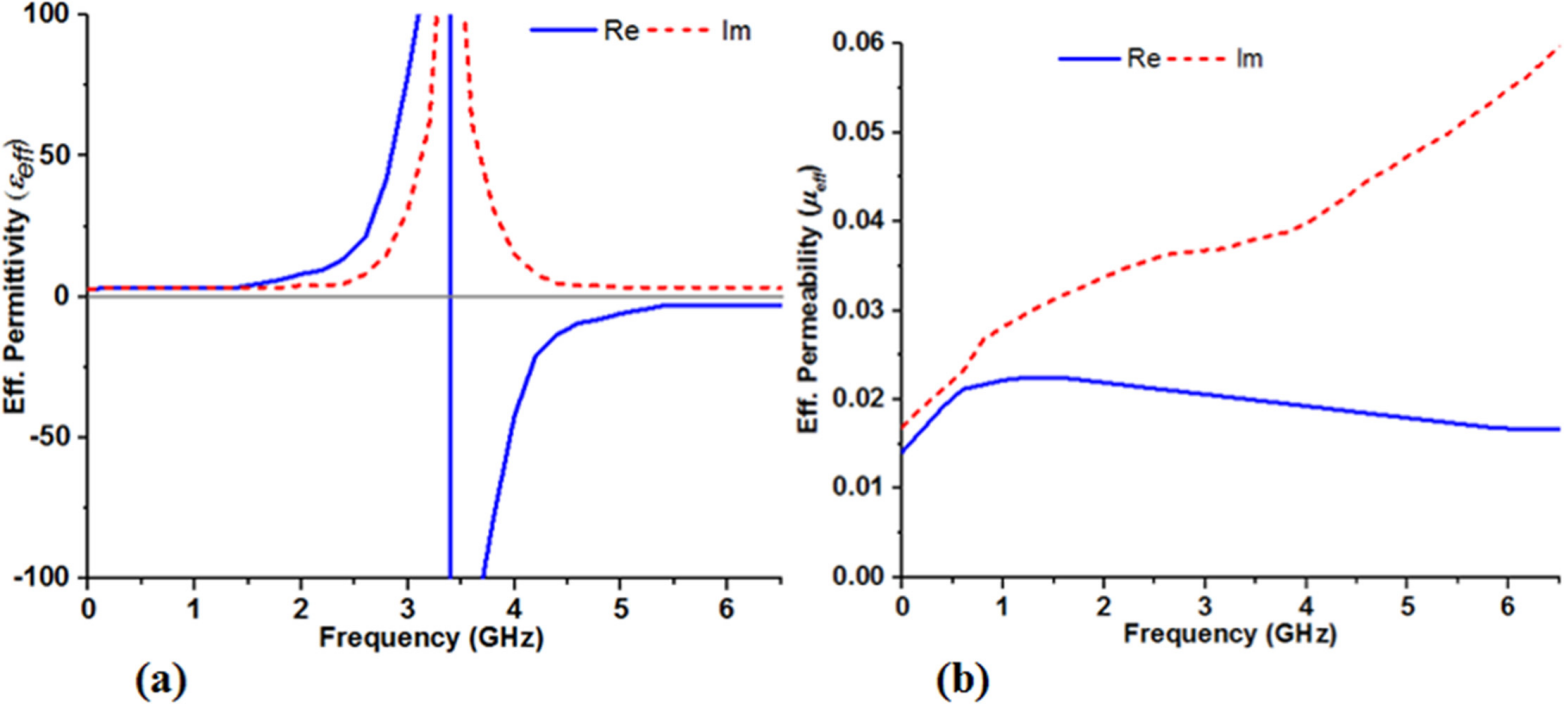
The retrieved constitutive parameters, (a) effective permittivity (*ε*_*eff*_) and (b) effective permeability (*μ*_*eff*_) of the proposed MSR structure.

For achieving better performance out of the conventional patch antenna, the design of the proposed MSR involves artificially engineering the metallic reflecting surface placed below the ground plane by exploiting the unwanted back radiating wave. For the designing of MSR structure, the idea of mesh grid arrays is employed which first implemented by French physicist Stefan Enoch [43]. The MSR structure for the designed antenna comprises of planar 4 x 5 array/matrix of square shaped element. The dimension of the square metallic element is 0.02λx0.02λ (with respect to the lowest resonant mode at 900 MHz) and a gap of 0.007λ is maintained between each of the elements. The graphical and geometrical view of the proposed metasurface structure is given in Fig 1 (B). The square-shaped element for various types of metasurface structures has been studied by the researchers around the world because of their excellent performance [44–46]. The 3D full-wave HFSS electromagnetic simulator is used on 64-bit operating system to predict the performance criteria and corresponding dimensions of the MSR embedded antenna. Through numerous simulation studies, the convergence has been reached by utilizing appropriate boundary conditions assigned to the overall design and setting up variable parameters to the Optimetrics engine of HFSS. Analyzing the antenna performance in terms of S-parameter, gain and radiation patterns, the optimized parameter value of each of the metasurface embedded antenna elements are determined with the help of the data retrieve from HFSS. The microwave interaction of the MSR structure can be examined by determining the effective constitutive parameters from the planar geometrical metasurface. Since the year 2000, some researchers have concentrated their work on easy and robust retrieval method for effective constitutive parameters (permittivity and permeability) to characterize the metamaterial and metasurface [13,15,47–49]. Most of the techniques are developed on the basis of utilizing the S-parameters (reflection and transmission coefficients) to first calculate the impedance *z* and refractive index *n*, and then the permittivity *ε* and permeability *μ* is being calculated. By studying the commonly used effective parameter extraction techniques, the effective constitutive parameters, permeability (*μ*_*eff*_) and permittivity (*ε*_*eff*_) are extracted; the graphical presentation is given in Fig 5. It is evident from the figure that the values of the retrieved parameters from the proposed MSR structure show remarkable behavior by posing the values for effective permeability in the range of 0 < *μ*_*eff*_ < 1. In a similar manner of applying general equation, the associated index of refraction *n* can be easily evaluated with the mathematical equation derived from Snell’s law, $n=\sqrt{\varepsilon_{eff}\times \mu_{eff}}$ [14]. So far, if the value of the effective permeability (*μ*_*eff*_) for the engineered metasurface structure becomes near to zero, following the equation the refractive index n approaches to zero as well. For a near zero-index material, incidence of plane wave on it transmitted in such a way that the refracted wave is directed along the normal to the interface. This phenomenon also suggests that the metasurface with very small values of index may still offer focusing the electromagnetic waves towards the broadside direction.

**Fig 6:**
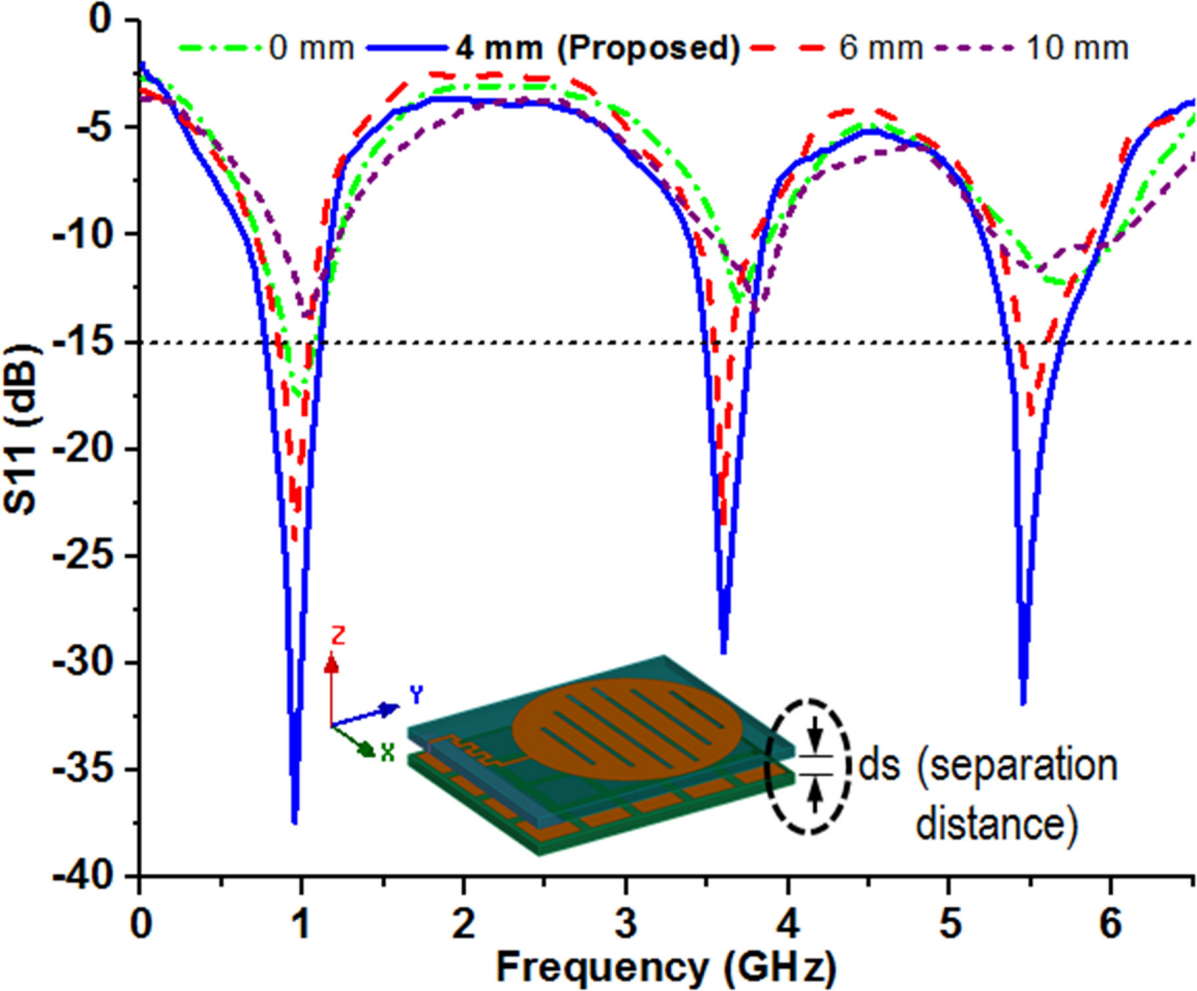
Effect of the distance of separation between the conventional microstrip patch antenna and MSR structure.

The metasurface is a 2D version of the bulky metamaterial structure which takes less physical space and offers a less lossy structure for possible artificial control of electromagnetic waves through it. As a new area of research, the cutting edge technology may lift up the designing and realization of metasurface towards novel functionalities and improved performance inclusion which may facilitate obtaining increased bandwidths and reduced losses. The fundamental intellection of the MSR structure design includes a periodic planar metallic layer that may help to reinforce the resonant excitation through electromagnetic reflection and thereby expansion of radiation aperture occurred. The periodic metallic surface interact with the impinging electromagnetic beam, the current is being induced within them though after a certain amount of metallic absorption. The optimized placement of the metallic elements resembles an inductive grid, whereas the space between them behaves as capacitive grid. Due to induced currents and combined effect of inductive-capacitive grid, the incident wave may face two possibilities; the negation of the phase of the incident wave or in phase distribution. In general, the repetitively positioned copper element produces in-phase currents with the original one from the microstrip patch antenna thus more directive emission can be occurred. This phenomenon makes the MSR as a finite ground plane to effectively reduce the back radiation and increases the radiation efficiency. The mutual coupling between the MSR structure and the microstrip antenna has been seriously considered and impedance matching in between them is investigated by varying the separation distance. The effect of separation height in the range of 0 to 10.0 mm is studied and their corresponding simulation results are presented through Fig 6. It can be observed that a separation distance of ds = 4.0 mm between the antenna and MSR structure offers adequate impedance bandwidth requirement by RFID and WLAN/WiMAX applications.

## Experimental Result Analysis

**Fig 7:**
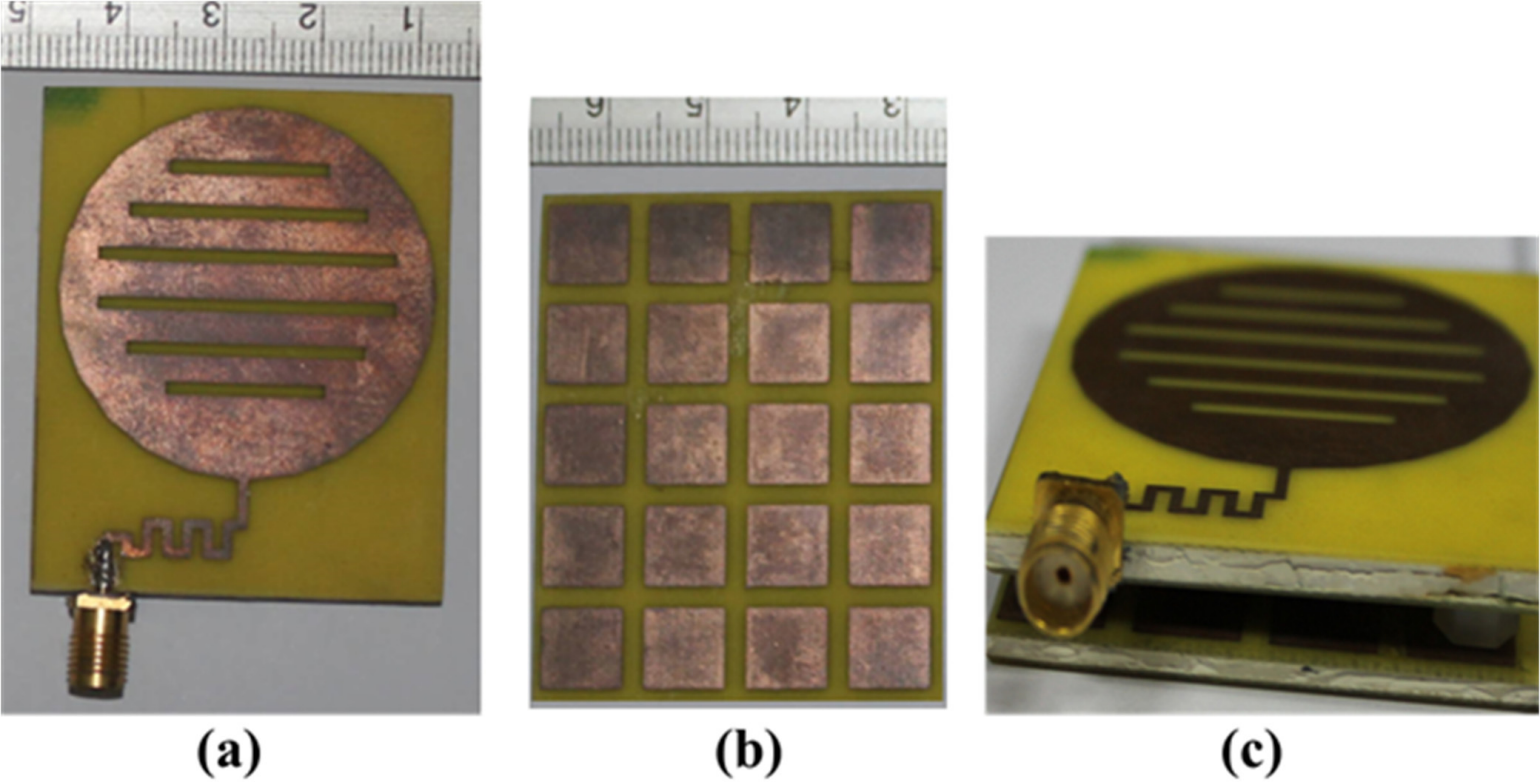
Photograph of the fabricated prototype (a) microstrip patch antenna, (b) MSR structure and (c) patch antenna with MSR.

The expected performances in terms of gain, S-parameter *S11*, radiation patterns of the triple band circular shaped slotted planar MSR embedded antenna have been investigated and numerically optimized with the help of electromagnetic structural simulation software HFSS, which generates the finite element model of Maxwell’s equations within volumetric boundary condition. Through numerous simultaneous simulations and applying optimization techniques using the Optimetrics tool integrated with HFSS, the optimal design has been achieved for the conventional patch antenna and MSR. To examine the actual performance and validate the simulation results obtained for the conventional antenna embedded with MSR, they are fabricated on ceramic filled bio-plastic substrate material of high dielectric constant as given in Fig 7.

**Fig 8:**
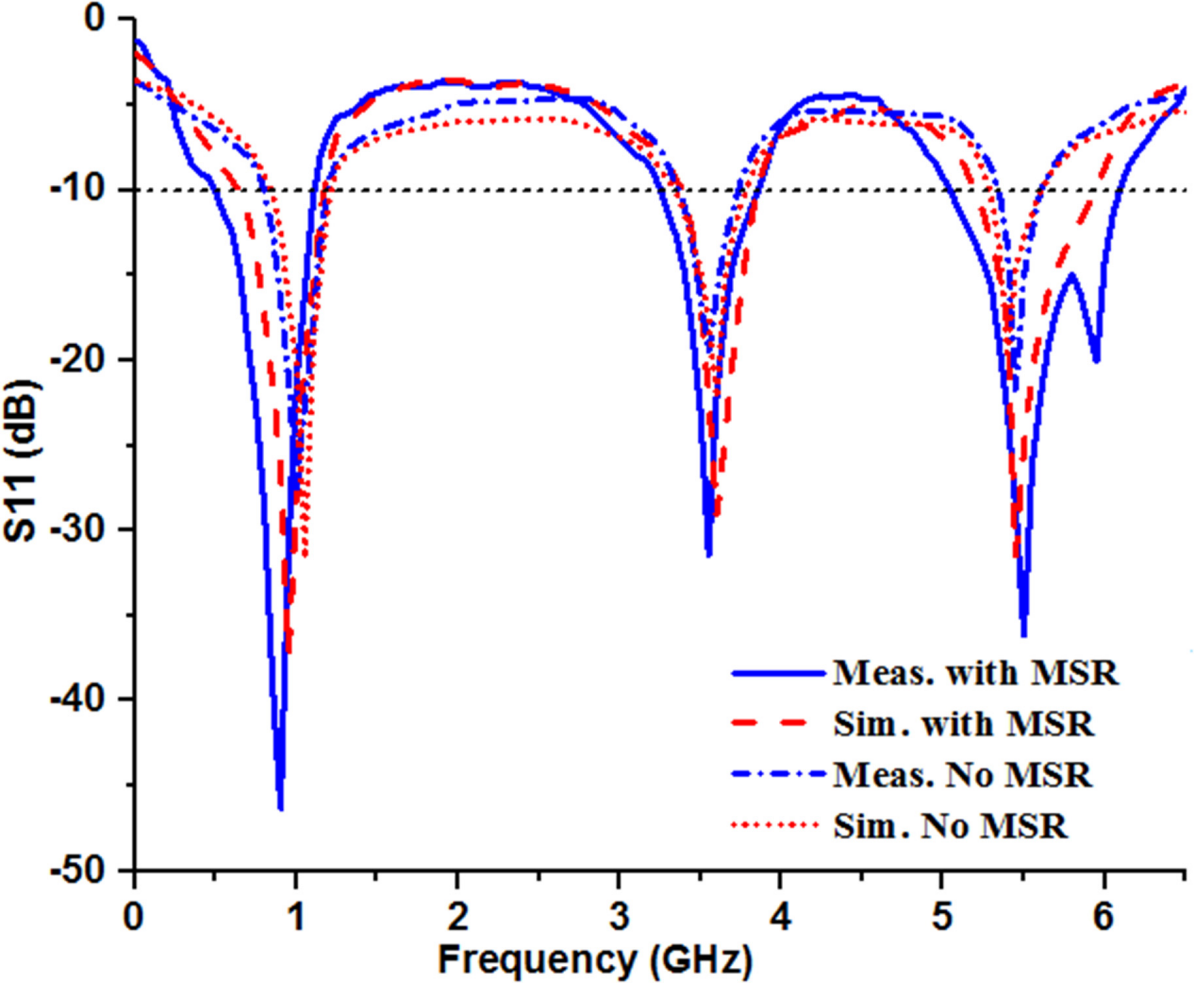
The measured and simulated S-parameter, *S11* of the antenna (with and without MSR integration).

The measurement of the conventional patch antenna and MSR loaded patch antenna have been performed in the standard anechoic chamber located at Microwave Laboratory, Faculty of Engineering and Built Environment, Universiti Kebangsaan Malaysia. An Agilent E8632C Precision Network Analyzer (PNA) was used for the entire measurement procedure. The Fig 8 shows the electromagnetic simulation and measurement results for S-parameter, *S11* against the microwave frequency, where good accordance can be seen in between them. However, still there are some differentiations which may be due to the coupling inconsistency between the SMA and radiating element, fabrication tolerance and/or interference effect from surrounding and various part of the antenna. From the obtained experimental results as depicted in Fig 8, three distinct operating bands can be seen while no MSR is used; 540 MHz (0.648–1.188 GHz), 467 MHz (3.364–3.831) and 758 MHz (5.185–5.943 GHz) with center frequency 0.937 GHz, 3.59 GHz and 5.44 GHz respectively. The figure clearly shows that the planar patch antenna loaded with MSR has certainly increased the operating bandwidths from 540 to 632 MHz (0.47–1.102 GHz), 467 to 606 MHz (3.25–3.856 GHz) and 758 MHz to 1062 MHz (5.045–6.107 GHz). Beside the meandered stripline feed and partial ground, the distant place of metallic periodic structure on a planar surface creates parasitic effects and electromagnetic coupling which may involve in widening the bandwidth. Embedding the MSR covers up the mutual coupling effect and surface wave by generating in-phase electromagnetic wave with the original excitation and thereby helps to improve the operating bandwidth and radiation efficiency.

**Fig 9:**
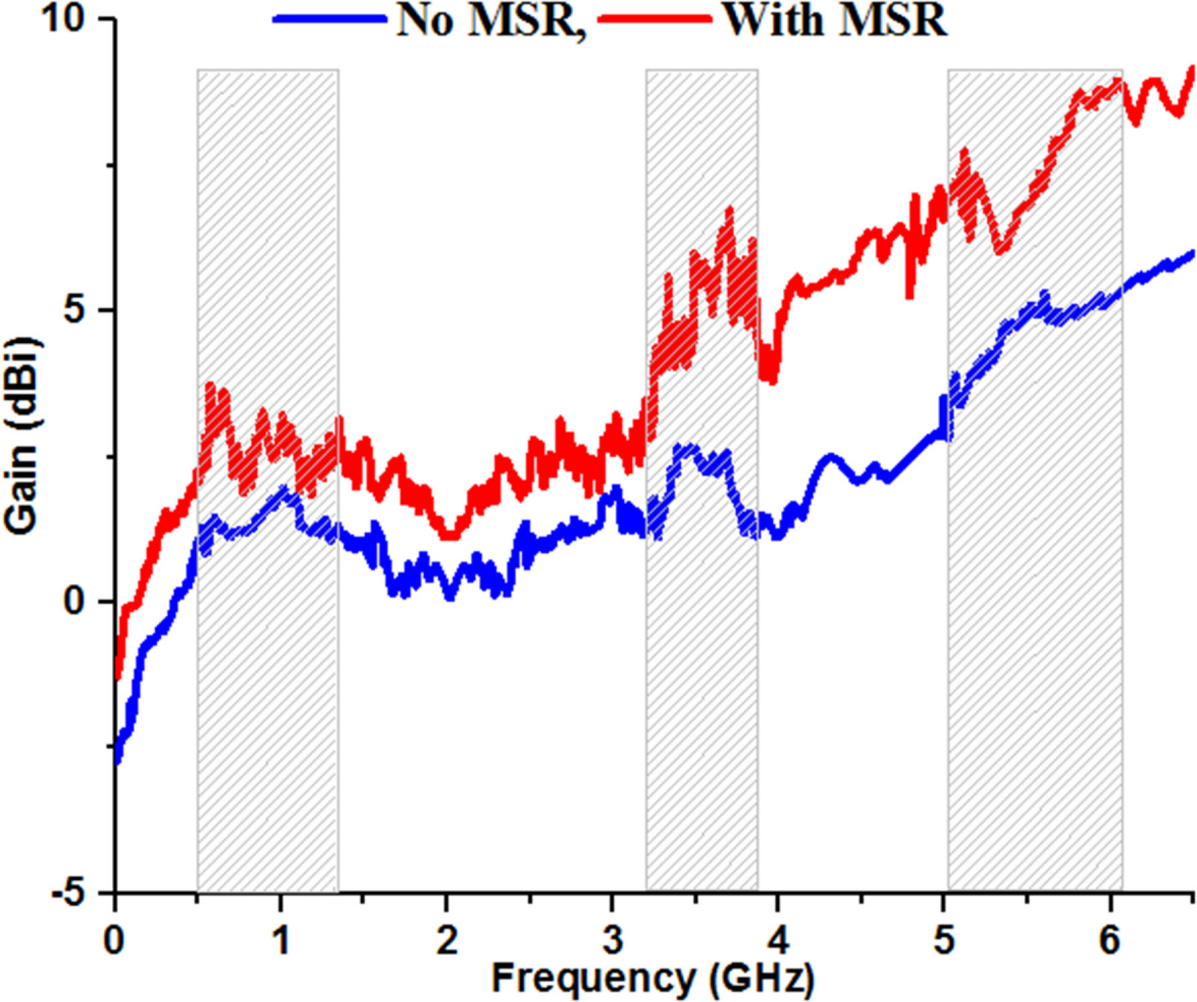
Measured gain of the proposed antenna with and without MSR structure.

The experimental gain of the proposed antenna between 0.1 GHz to 6.5 GHz is plotted in Fig 9, where MSR incorporated microstrip antenna is compared to the conventional microstrip antenna. The standard three antenna measurement technique has been employed for the evaluation of gain. Inside the standard anechoic chamber, the two identical horn antennas of known parameters are used as reference antenna for the antenna under test measurement. As from the figure it can be noticed that the integration of MSR structure to the conventional microstrip antenna has substantially increased the gain as expected. The increment of gain may be resulted from the electromagnetic coupling between the MSR structure and radiating patch, and cavity effect. The patch antenna with optimized position of MSR structure have achieved enhanced gain from 1.98 dBi to 3.02 dBi, 2.58 dBi to 5.92 dBi and 4.57 dBi to 8.47 dBi in the lower, middle and upper band respectively. The gain of the conventional patch antenna can be further improved by increasing the numbers of MSR element. However, the overall volumetric size of the antenna will be increased which severely affect the compactness of the total structure.

**Fig 10:**
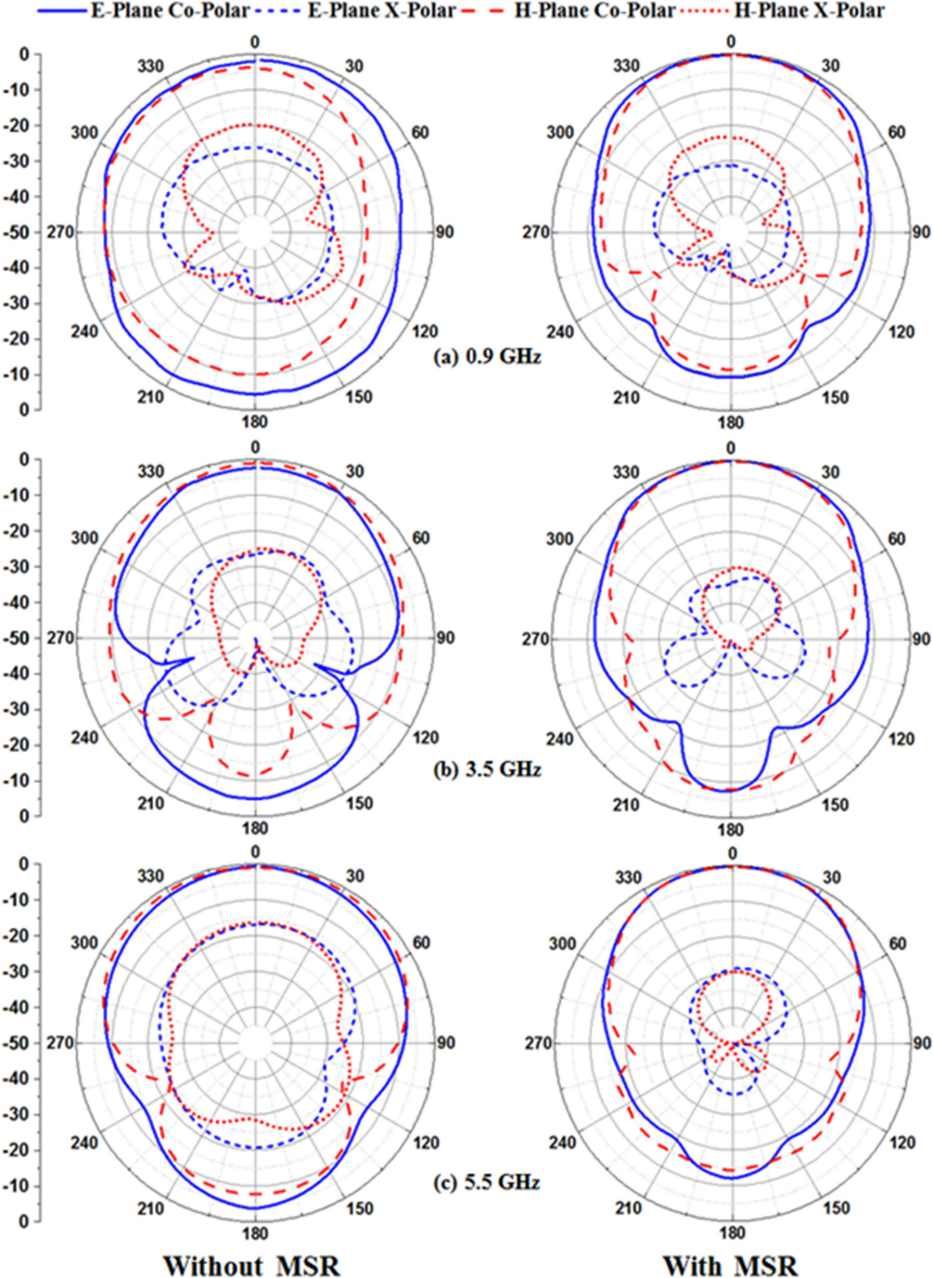
The simulated radiation pattern of the proposed antenna without and with MSR structure at three different resonant frequencies.

**Fig 11:**
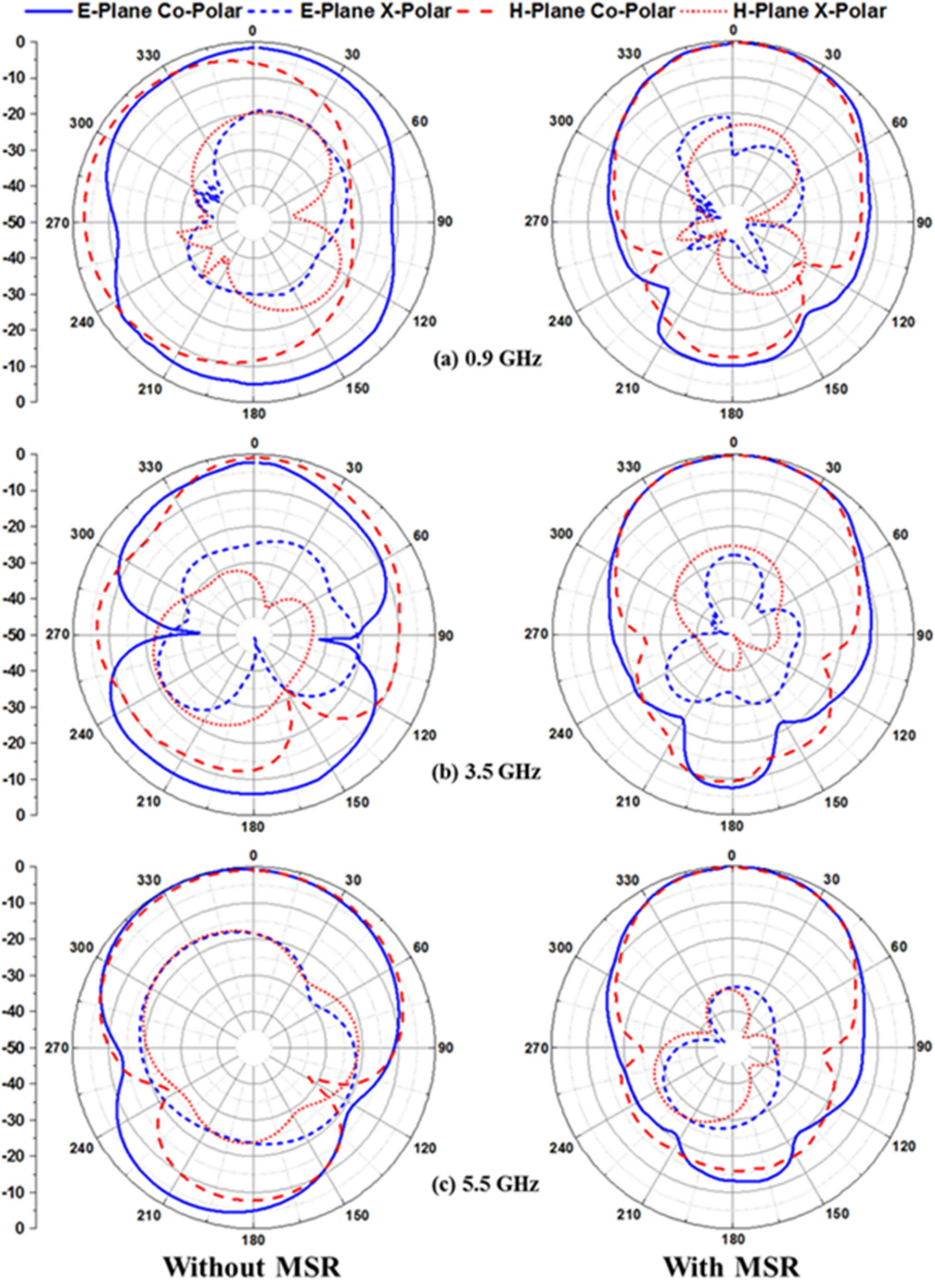
The measured radiation pattern of the proposed antenna without and with MSR structure at three different resonant frequencies.

**Fig 12:**
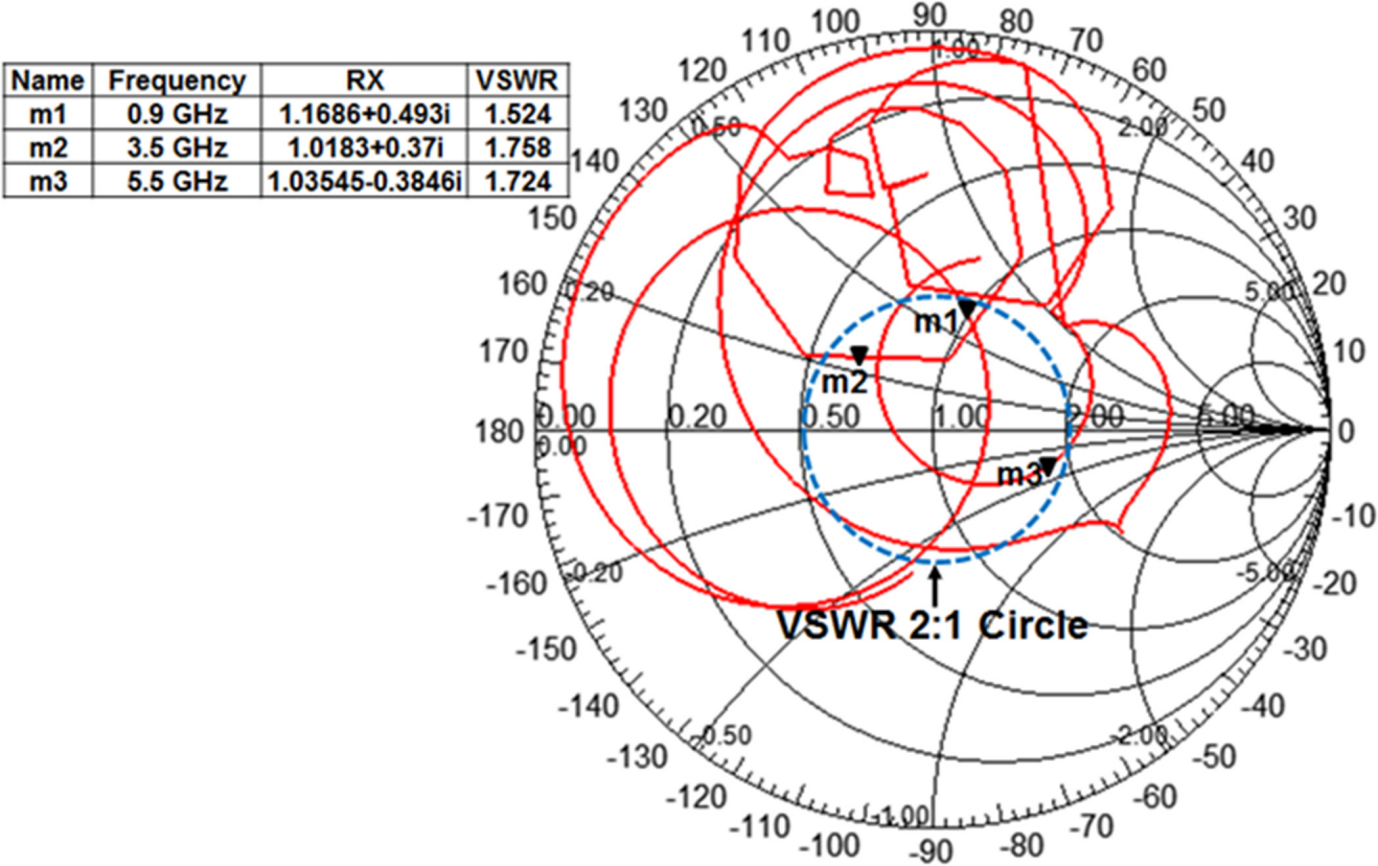
Smith chart of the proposed MSR loaded microstrip patch antenna.

The simulated normalized radiation patterns at 0.9 GHz, 3.5 GHz and 5.5 GHz in E- and H-plane are illustrated in Fig 10. The profiles for measured radiation patterns of the proposed microstrip patch antenna with and without the MSR structure at 0.9 GHz, 3.5 GHz and 5.5 GHz in E- and H-plane are normalized and depicted in Fig 11. It is observed from the figure that besides the improvement of bandwidth and gain, the proposed MSR embedded patch antenna shows excellent broadside radiation with substantial directional radiation properties. Moreover, the back lobe radiation pattern is slightly reduced; however, still there are some effects due to the partial ground plane and cable loss in the standard system of measurement that leads to spurious back radiation. Further investigation on the proposed multiband microstrip patch antenna with MSR structure is performed in terms of input impedance and voltage standing wave ratio (VSWR) as presented through the Smith chart in Fig 12. The figure easily delivers the information that the working frequency bands for UHF RFID and WLAN/WiMAX reside within the VSWR 2:1 circle. The resonant frequencies are marked as *m1*, *m2* and *m3*, and the values for VSWR, input impedance *Rx* are listed in the table.

## Conclusion

This paper introduces a simple design of microstrip patch antenna embedded with the MSR structure for enhancing the bandwidths, gains and radiation patterns simultaneously, cater the services for UHF/microwave (5.8 GHz) RFID, 3.5/5/5 WiMAX and 5.2/5/8 GHz WLAN communication bands. The proposed simple designed planar microstrip patch antenna loaded with MSR is fed by optimized meander stripline and backed by partial ground plane. Beside the achievement of significant gain and radiation pattern improvement by reason of incorporating MSR structure, it helps to attain wide enough working bandwidth of 632 MHz (0.47–1.102 GHz), 606 MHz (3.25–3.856 GHz) and 1062 MHz (5.045–6. 107 GHz) which resonates at 0.9, 3.54 and 5.5 GHz respectively. The key parameters for designing, tuning and analyzing the antenna structure and its performance criteria are discussed in the paper. The experimental and numerical results for S-parameter *S11*, peak gains, and radiation patterns affirm reasonable performance and good agreement between them. Therefore, the proposed MSR integrated microstrip antenna can be a good candidate to offer the services for RFID, WiMAX/WLAN applications.
